# Transcriptomic analysis of human primary T cells after short-term leucine-deprivation and evaluation of kinase GCN2’s role in regulating differential gene expression

**DOI:** 10.1371/journal.pone.0317505

**Published:** 2025-02-18

**Authors:** Aurore Dougé, Gwendal Cueff, Céline Keime, Valérie Carraro, Céline Jousse, Paul Rouzaire, Alain Bruhat

**Affiliations:** 1 Service d’oncologie médicale, CHU Gabriel Montpied, Clermont-Ferrand, France; 2 Université Clermont Auvergne, EA Chelter 7453, Clermont-Ferrand, France; 3 Université Clermont Auvergne, INRAE, UNH, UMR1019, Clermont-Ferrand, France; 4 IGBMC - Institut de Génétique et de Biologie Moléculaire et Cellulaire, Illkirch, France; 5 Université Clermont Auvergne, Clermont-Ferrand, France; 6 Service d’histocompatibilité et d’immunogénétique CHU Gabriel Montpied, Clermont-Ferrand, France; University of Wisconsin-Milwaukee, UNITED STATES OF AMERICA

## Abstract

Chimeric Antigen Receptor T (CAR-T) cells offer a promising strategy for cancer treatment. These CAR-T cells are either autologous or allogeneic T cells that are genetically modified to express a chimeric antigen receptor targeting a specific tumor antigen. Ongoing research aims to optimize the CAR-T cell efficacy, including strategies to modulate their metabolism. One such approach involves inducing transgene expression by activating the GCN2 kinase signaling pathway through dietary deprivation of an essential amino acid. In this study, we investigated the general impact of a 6-hour leucine deprivation on primary activated human T cells using RNA-seq technology. Our analysis identified 3,431 differentially expressed genes between T cells cultured in regular medium and those cultured in leucine-deprived medium. Gene Set Enrichment Analysis revealed that “TNFα signaling via NFκB”, “interferon-γ response”, and “unfolded protein response” gene sets were positively enriched, while “mTORC1 signaling”, “Myc targets”, and “oxidative phosphorylation” gene sets were negatively enriched. To further evaluate the involvement of GCN2 kinase in regulating the differential gene expression during the 6-hour leucine deprivation, T cells were cultured with or without a GCN2 inhibitor. We found that 59% of the differentially expressed genes in our dataset were dependent on the kinase GCN2 (n = 2028), with 1,140 up-regulated and 888 down-regulated genes. These findings suggest a promising strategy to enhance CAR-T cell efficacy by combining short amino acid starvation with transient overexpression of a target gene.

## Introduction

Cell therapy is a therapeutic approach that utilizes immune system cells to replace, repair, enhance, or modify the biological activity of damaged tissues or organs. This approach is widely employed in the treatment of leukemia, particularly through allogeneic hematopoietic stem cell transplantation (allo-HSCT) and more recently, Chimeric Antigen Receptor T cells (CAR-T). CAR-T cells are currently autologous T lymphocytes harvested from the patient, which are modified *ex vivo* to express a chimeric antigen receptor specific to a tumor antigen of interest. These lymphocytes are then reinfused into the patient, where they can self-activate upon recognizing their target antigen, directly destroying tumor cells that carry the antigen. CAR-T cells targeting CD19 and BCMA have been granted marketing authorization for the treatment of certain hematological malignancies that are refractory to conventional chemotherapy. The overall development of CAR-T cells is progressing rapidly, including for the treatment of solid tumors [1]. However, this innovative therapeutic approach has areas for improvement, particularly in terms of efficacy, as a significant percentage of patients may experience a decline in the competence of their CAR-T cells over time, potentially due to T cell exhaustion [2]. Consequently, numerous studies are ongoing to optimize the CAR-T cell strategy [1].

Among these approaches, one focuses on modulating the metabolic phenotype of CAR-T cells, since it significantly impacts their function, differentiation, and persistence. As a result, many strategies are being explored to target CAR-T cell metabolism, including the optimization of cell culture conditions, modulation of glycolysis or oxidative phosphorylation, promotion of mitochondrial biogenesis, and nutritional interventions [3–5]. There are also numerous challenges in controlling CAR-T cells once they are reintroduced into the patient, either to enhance their therapeutic action or to limit their toxicity.

In this context, we are currently investigating the NUTRIREG system, that exploits the GCN2-ATF4 pathway’s ability to induce transgene expression following the consumption of a diet deficient in an Essential Amino Acid (EAA) [6]. The initial step of this pathway is the activation of the GCN2 kinase by uncharged tRNAs. GCN2 then phosphorylates the α subunit of eukaryotic initiation factor 2 (eIF2 α) on serine 51, leading to upregulation of the translation of the activating transcription factor 4 (ATF4). Once induced, ATF4 activates transcription of many specific target genes through binding to Amino Acid Response Elements (AARE).

NUTRIREG is based on the combination of (i) an artificial promoter derived from AARE sequences, that is highly inducible by EAA deficiency and controls the expression of a transgene of interest and (ii) a diet deficient in one EAA, leading to a significant decrease in the blood concentration of the limiting EAA and activation of the GCN2/ATF4 signaling pathway. After delivering the AARE-Gene plasmid to a target tissue using a viral vector, transgene expression can be induced upon consumption of an EAA-deficient diet. Interestingly, this pathway can be rapidly turned off by consuming the deficient EAA, providing an easily reversible regulation system devoid of adverse effects, as it operates through a physiological nutritional pathway. After validating that the GCN2-ATF4 pathway can be induced in human T cells through culture in an EAA-deficient medium, we demonstrated the proof of concept for the functionality of NUTRIREG in T cells using eGFP and luciferase reporter genes, as well as the gene encoding IL-10 [7]. We propose that NUTRIREG could be a tool of interest to reprogram the metabolic activity of T cells after infusion to improve their antitumor efficacy, longevity, and function. Before exploiting the therapeutic potential of NUTRIREG in the field of cell therapy with short-term EAA deprivation, we here investigated the general impact of 6-hour leucine deprivation and the role of GCN2 on primary activated human T cells using RNA-seq technology.

## Materials & methods

### Preparation of human primary T cells

PBMCs were purified from buffy coats collected from two different healthy donors by the French Blood Establishment (EFS - Etablissement Français du Sang) using Cytiva Ficoll-Paque^TM^ PLUS (Fisher). In a second step, human T cells were isolated from donors PBMCs using the human Pan T Cell Isolation Kit (Miltenyi Biotec, Germany) according to the manufacturer’s instructions. Isolated T cells were then resuspended in RPMI 1640 medium (PAN BIOTECH) at a density of 1-2x10^6^ cells/ml. Purified human T cells were activated using a human T-cell Activation/Expansion Kit (Miltenyi Biotec, Germany). The ratio of MACSiBead^TM^ particles per cell was 1:2. RPMI medium containing 10% heat-inactivated FBS was supplemented with human IL-2 IS (Miltenyi Biotec, Germany) at 50 IU/ml. T cells from donor 1 were used for the RNA-Seq analysis, whereas T cells from donor 2 were used for the RT-qPCR analysis.

### T cell culture and treatments

Activated human T cells were incubated in 10% FBS-RPMI medium supplemented with IL-2 at 37°C and 5% CO2 for 7 days of expansion. At day 8 post activation, Dulbecco’s Modified Eagle’s Medium (DMEM) (with 10% of dialyzed calf serum) lacking leucine was used (GENAXXON bioscience). A control medium was simultaneously reconstituted by adding all nine EAAs. RPMI-cultured human T cells were collected and washed in PBS with centrifugation at room temperature at 300xg for 5 min. Cells were then resuspended either in reconstituted DMEM control or in DMEM lacking leucine, at a density of 1x10^6^ cells/mL. DMEM was freshly supplemented with IL-2 (50 IU/mL). GCN2 inhibitor (TAP20) was previously described [7–9] and generously provided by Merck KGaA. TAP20 is a (1R,3R)-3-[3-(2-methyl-quinolin-6-yl)-3H-[1,2,3]triazolo[4,5-d]pyrimidin-5-ylamino]-cyclopentanecarboxylic acid amide and is known to exhibit high potency and selectivity against GCN2. It has been demonstrated that TAP20 strongly suppressed ATF4 expression when used at concentration>  1uM [10]. We evaluated the effectiveness of this GCN2 inhibitor in suppressing the mRNA expression of the TRB3 gene (an ATF4 target gene) at different concentrations (1, 2.5, and 5 μM) during 24 hours of leucine deprivation and observed inhibition of TRB3 expression starting at 1 μM (data not shown). We chose to use the intermediate concentration of 2.5 μM to ensure there was no activation of the eIF2α-ATF4 pathway *via* GCN2. We then validated that the inhibition of TRB3 (RT-qPCR) and ATF4 (western blot) was indeed effective during 6 hours of leucine deprivation with TAP20 at a concentration of 2.5 μM [7]. TAP20 was then resuspended in DMSO and used at a final concentration of 2.5 µ M. Cells were cultured in DMEM Control or DMEM lacking leucine + /- GCN2 inhibitor for 6 hours and were then collected for mRNA extraction. T cells culture and treatment were performed under the same conditions but independently for each of the two donors. The replicates (n = 4) are technical replicates of the samples derived from each of the two donors.

### mRNA extraction

mRNA of each sample was extracted with a NucleoSpin RNA kit (Macherey Nagel) according to the manufacturer’s instructions. mRNA quality was validated by Nanodrop analysis. mRNA samples were stored at -80°C until gene expression analysis.

### Real-time quantitative PCR

Real-time RT-qPCR was performed as previously described [11]. Primers for the human sequences yielded PCR products of approximately 100–150 bp in size. The abundance of each mRNA was normalized to the β-actin signal.

### RNA-seq analysis

RNA sequencing was performed by the GenomEast platform, member of the ‘France Génomique’ consortium (ANR-10-INBS-0009). RNA-Seq libraries were generated from 500 ng of total RNA using TruSeq Stranded mRNA Library Prep Kit and TruSeq RNA Single Indexes kits A and B (Illumina, San Diego, CA), according to manufacturer’s instructions. Libraries were sequenced on an Illumina Hiseq4000 sequencer as single end 50 bases reads. Reads were preprocessed to remove the adapter, polyA, and low-quality sequences (Phred quality score below 20). After this preprocessing, reads shorter than 40 bases were discarded for further analysis. These preprocessing steps were performed using cutadapt [12] version 1.10. Reads were mapped to rRNA sequences using bowtie [13] version 2.2.8 and reads mapping to rRNA sequences were removed for further analysis. Reads were mapped onto the hg38 assembly of the Homo sapiens genome using STAR [14] version 2.5.3a. Gene expression quantification was performed using htseq-count [15] version 0.6.1p1, with annotations from Ensembl version 99 and “union” mode. Only non-ambiguously assigned reads have been retained for further analyses. Raw and processed data have been recorded on GEO (GSE279263).

### Differential gene expression analysis

The raw counts data as released by the sequencing platform were processed under R software (v4.3.0) thanks to edgeR library (v4.0.9). Overall, edgeR was used to filter out genes with a global low expression and to perform a TMM (Trimmed Mean of M-values) data normalization, to compensate experimental bias. The differential expression analysis was performed with the R package limma (v3.58.1) with its voom method. In limma, the Bonferroni method was chosen to adjust the p-values for multiple testing and a threshold at 0.05 was the cutoff for significant genes. For these significant genes, the CLD (Compact Letter Display) profiles were calculated thanks to lsmeans package functions (v2.30.0), to help interpret the expression variations between experimental groups. Here log-cpm transformed counts data were used to switch from a discrete context to a continuous one. As a multivariate approach, a PCA analysis was applied to the same transformed data with two R librairies: FactoMineR (v2.11) and factoextra (v1.0.7) to get individuals and variables plots. In this analysis, the data quality and the main biological effects were assessed. For genes sets comparison, seeking for specific or common genes, instead of the classical venn diagrams, the upsetR library (v1.4.0) enabled barplot visualisations. Gene Ontology and Gene Set Enrichment Analysis was performed using BigOmics analytics (https://bigomics.ch/).

### Ethics statement

Primary T cells used in this research project come from human blood products and its components for non-therapeutic purposes, obtained under a transfer agreement signed with the French Blood Establishment (agreement 19-088). Human blood products and its components for non-therapeutic use refer to all products derived from blood collection performed by the blood transfusion establishment, regardless of their packaging, and intended for laboratory research, or the manufacturing of *in vitro* diagnostic medical devices, or teaching purposes, or for conducting medical biology tests and analyses, excluding any therapeutic use. The donors signed the donor consent collection form during a pre-donation interview. Auvergne-Rhônes-Alpes French Blood Establishment has the authorization from the French Ministry of Education for preservation and preparation activities for scientific purposes of elements derived from the human body (CODECOH AC-2020-3959).

## Results

**Fig 1:**
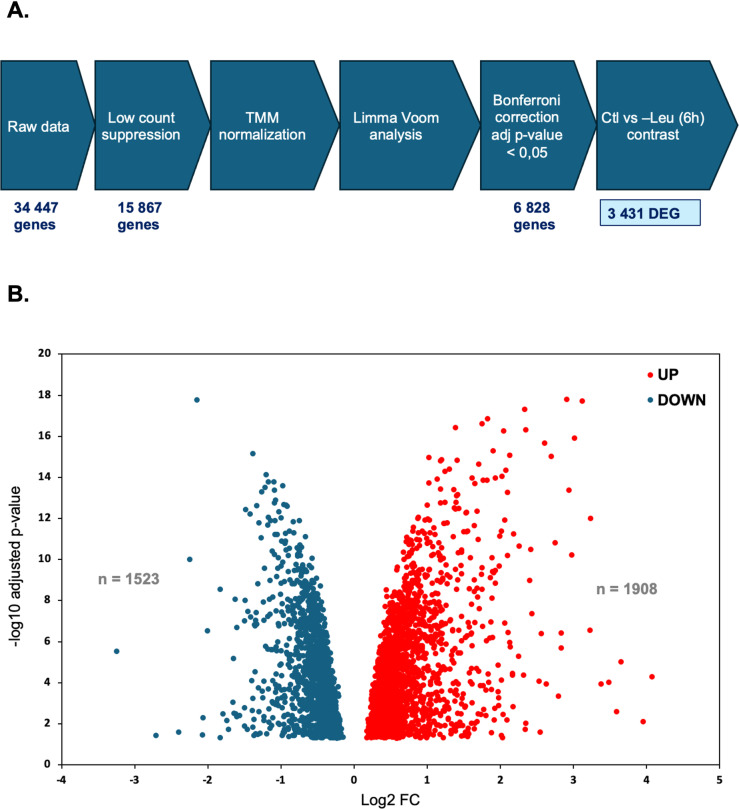
Identification of the differentially expressed genes (DEGs) between T cells cultured in control media and T cells cultured in leucine-deprived media for 6 hours. **A** Bioinformatic methodology employed to select significant DEGs (adjusted p-value <  0.05; fold-change FC < or >  1). **B.** Volcano-plot displaying fold-change (Log2-FC) versus significance (-Log10 p-value). Red circles represent up-regulated genes (Log2-FC >  0) and blue circles represent down-regulated genes (Log2-FC <  0).

Human activated T cells were cultured in DMEM Control or in DMEM lacking leucine for 6 hours before being collected for mRNA extraction and then RNA-seq analysis. After bioinformatic processing of raw data (Fig 1A), we identified a total of 3,431 differentially expressed genes (DEGs) (adjusted p-value <  0.05) between T cells cultured in control media and T cells cultured in leucine-deprived media for 6 hours. The volcano plot, which displays fold-change versus significance of these DEGs, showed that 1,908 genes were upregulated after 6 hours of leucine deprivation (fold-change >  1 compared to control media), while 1,523 genes were downregulated (fold-change <  1) (Fig 1B). The lists of the 50 most upregulated genes (fold-change ≥  4.09) and the 50 most downregulated genes (fold-change ≤  0.41) are presented in S1 Table.

**Fig 2:**
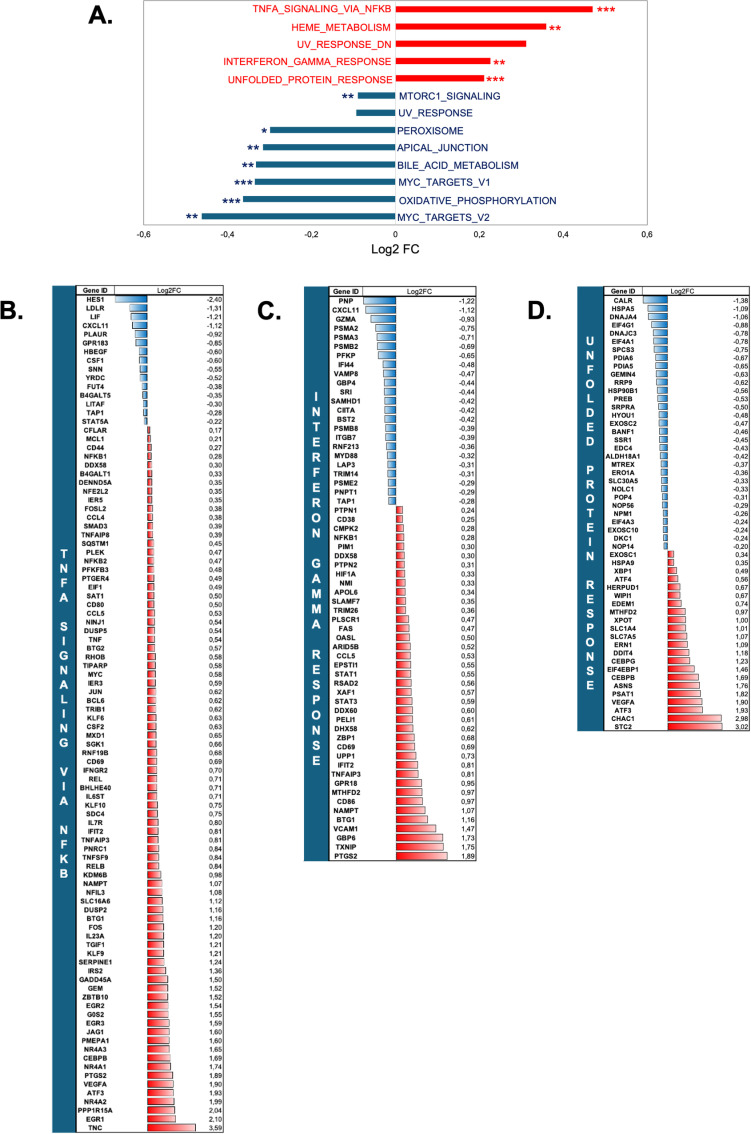
Gene Set Enrichment Analysis (GSEA) of T cells after culture in leucine-deprived media for 6 hours. **A** GSEA using the Molecular Signatures Database (MSigDB) hallmark gene set collection. Results of gene set enrichment are expressed in Log2-FC. The number of stars indicates enrichment significance according to fgsea q-value ( * <  0.05, ** <  0.01, *** <  0.001). Red bars represent positively enriched gene sets (log2-FC >  0) and blue bars represent negatively enriched gene sets (log2-FC <  0). **B.** mRNA expression level of DEGs in the “TNFα signaling via NFκB” gene set. **C.** mRNA expression level of DEGs in the “interferon-γ response” gene set**. D.** mRNA expression level of DEGs in the “unfolded protein response” gene set**. B-D.** Results are expressed as a mRNA log2-fold-change between leucine-free (6h) and control media. Red bars represent up-regulated genes (log2-FC >  0) and blue bars represent down-regulated genes (log2-FC <  0).

We performed gene set enrichment analysis (GSEA) using the Molecular Signatures Database (MSigDB) hallmark gene set collection. Each hallmark gene set in this collection is a “refined” set, derived from multiple “founder” sets, that represents a specific biological state or process and exhibits coherent expression patterns [16]. Our results showed positive enrichment for the “TNFα signaling via NFκB” (fgsea q-value =  0.0006), “interferon-γ response” (q-value =  0.004), and “unfolded protein response” (q-value =  0.0006) gene sets, whereas “mTORC1 signaling” (q-value =  0.007), “Myc targets” (q-value V1 =  0.0006, and V2 =  0.002), and “oxidative phosphorylation” (q-value =  0.0006) gene sets were negatively enriched (Fig 2A). The proportion of DEGs in our dataset within positively enriched gene sets was 96/200 for the “TNFα signaling via NFκB” gene set, 63/200 for the “interferon-γ response” gene set, and 55/113 for the “unfolded protein response” gene set, with the ratio of upregulated genes being 84%, 63%, and 43%, respectively, in each of the three gene sets (Figs 2B-D). We validated the mRNA expression levels of 12 genes identified from RNA-Seq analysis by RT-qPCR in T cells collected from another healthy donor (S1 Fig).

**Fig 3:**
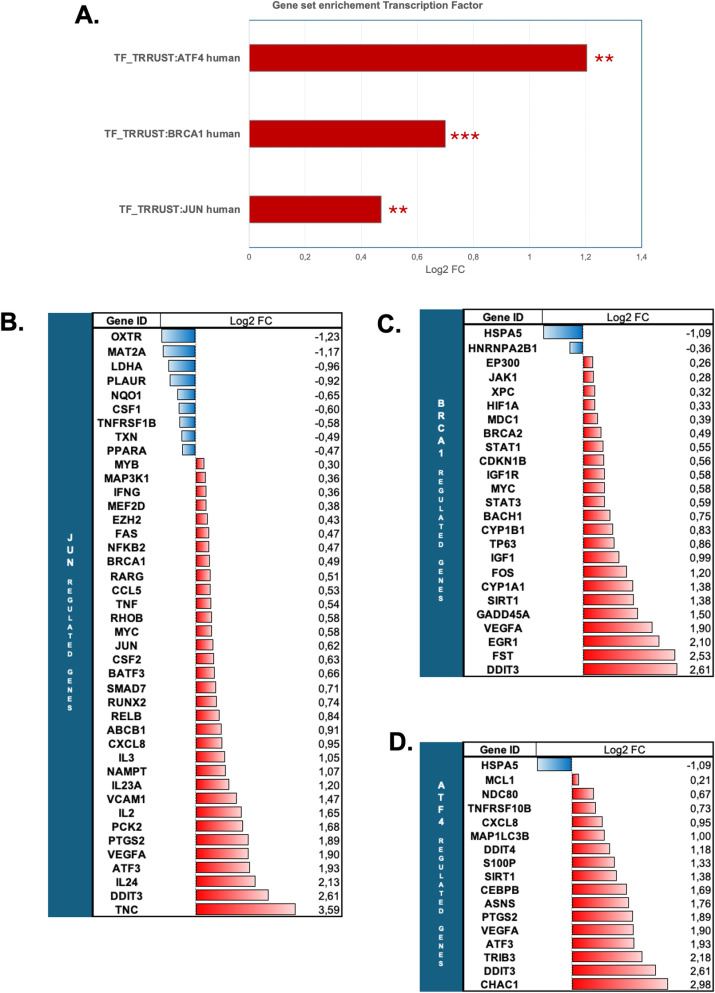
Transcription factor gene set enrichment analysis of T cells after culture in leucine-deprived media for 6 hours. **A** TF**-**GSEA using TRRUST (Transcriptional Regulatory Relationships Unraveled by Sentence-based Text mining). Results of gene set enrichment are expressed in Log2-FC. The number of stars indicates enrichment significance according to meta q-value (** <  0.01, *** <  0.001). Red bars represent positively enriched gene sets (log2-FC >  0). **B.** mRNA expression level of DEGs in the JUN-regulated gene set. **C.** mRNA expression level of DEGs in the BRCA1-regulated gene set**. D.** mRNA expression level of DEGs in the ATF4-regulated gene set**. B-D.** Results are expressed as a mRNA log2-fold-change between leucine-free (6h) and control media. Red bars represent up-regulated genes (log2-FC >  0) and blue bars represent down-regulated genes (log2-FC <  0).

We also performed gene set enrichment analysis for transcription factors in our dataset. TRRUST (Transcriptional Regulatory Relationships Unraveled by Sentence-based Text mining) analysis revealed significant positive enrichment of three transcription factor gene sets: JUN (Log2-FC =  0.47; q-value =  0.0048), BRCA1 (Log2-FC =  0.699; q-value =  0.0006), and ATF4 (Log2-FC =  1.204; q-value =  0.0014) (Fig 3A). The proportion of DEGs in these gene sets was 42/149 for the JUN gene set, 25/57 for the BRCA1 gene set, and 17/35 for the ATF4 gene set, with the ratio of upregulated genes being 79%, 92%, and 94%, respectively, in each of the three gene sets (Figs 3B-D).

**Fig 4:**
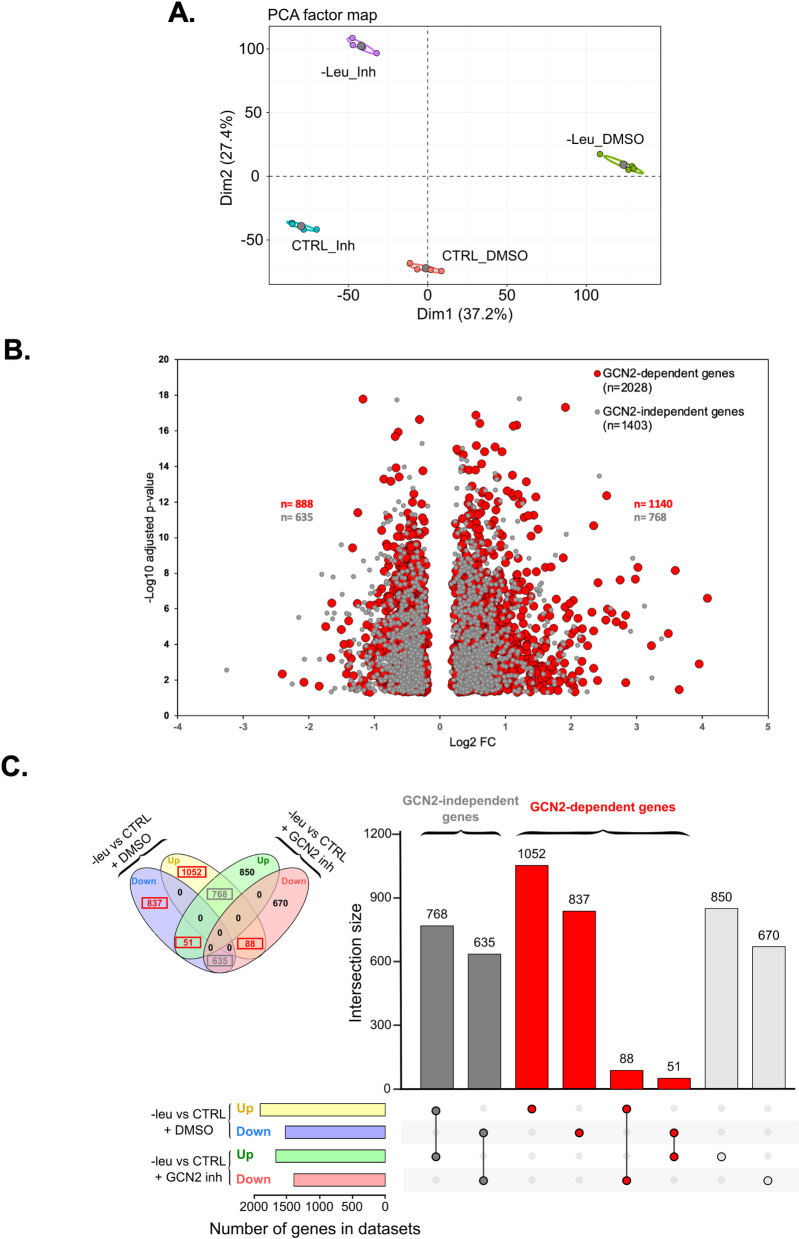
Identification of the DEGs between T cells cultured in control media and T cells cultured in leucine-deprived media for 6 hours with or without GCN2 inhibitor. **A** Principal component analysis. C_DMSO represents the samples of T cells cultured in control media, Leu_DMSO represents the samples of T cells cultured in leucine-free media during 6h, C_Inh represents the samples of T cells cultured in control media with 2.5uM of GCN2 inhibitor, Leu_Inh represents the samples of T cells cultured in leucine-free media with 2.5uM of GCN2 inhibitor during 6h. **B.** Volcano-plot displaying fold-change (Log2-FC) versus significance (-Log10 p-value) according to GCN2-dependency. Red circles represent GCN2-dependent genes and grey circles represent GCN2-independant genes. **C.** UpSetR plot of the intersections of sets of DEG (up or down) in the 2 culture conditions (+DMSO or +  GCN2 inhibitor). Each dataset is shown at the left of the matrix as bar chart showing the size (number of gene) of the dataset. For each set of DEG filled circles connected by a black line are placed in the corresponding matrix cell. If a set is not part of the intersection, a light gray circle is shown. Intersections showing genes that are GCN2-independent are shown in dark grey, and intersections showing genes that are GCN2-dependent are shown in red. The size (number of genes) of each intersection is shown as a bar chart placed on top of the matrix. The same intersections are shown as a classical Venn diagram as an insert at the top left of the figure with matching color code.

To assess the role of GCN2 kinase in the modulation of gene expression during 6 hours of leucine deprivation, T cells were cultured with or without a GCN2 inhibitor. Principal component analysis of RNAseq data identified two axes that explained 64.6% of the variance between the sample groups (37.2% by axis 1 and 27.4% by axis 2) (Fig 4A). We observed that the two leucine-deprivation groups (-Leu (6h) and -Leu (6h) +  GCN2 Inh.) were distinct from the two control groups (Ctl and Ctl +  GCN2 Inh.), demonstrating the impact of leucine deprivation on gene expression. These two -Leu groups (with or without GCN2 inhibitor) were also distinct from each other, indicating that one group of genes had their expression altered due to GCN2 kinase activation (defined as GCN2-dependent genes), while another group of genes experienced changes in expression due to leucine deprivation independent of GCN2 kinase (defined as GCN2-independent genes) (Fig 4A). We then applied Venn Contrast analysis on our dataset to categorize genes according to GCN2 dependency. The volcano plot displaying fold-change versus significance showed that 59% of DEGs in our dataset were dependent on GCN2 kinase (n = 2028), with 1,140 upregulated and 888 downregulated GCN2-dependent genes (Fig 4B). We defined as GCN2-dependent, genes that were (i) significantly upregulated by leucine deprivation and significantly downregulated by leucine deprivation with GCN2 inhibitor, (ii) significantly downregulated by leucine deprivation and significantly upregulated by leucine deprivation with GCN2 inhibitor, (iii) significantly up or downregulated by leucine deprivation with no significant differential expression in the presence of a GCN2 inhibitor. We defined as GCN2-independent, genes that were (i) significantly upregulated by leucine deprivation and still significantly upregulated by leucine deprivation with GCN2 inhibitor, (ii) significantly downregulated by leucine deprivation and still significantly downregulated by leucine deprivation with GCN2 inhibitor (Fig 4C). We validated the impact of the GCN2 inhibitor on mRNA expression levels of 12 genes by RT-qPCR in T cells collected from another healthy donor (S2 Fig).

**Fig 5:**
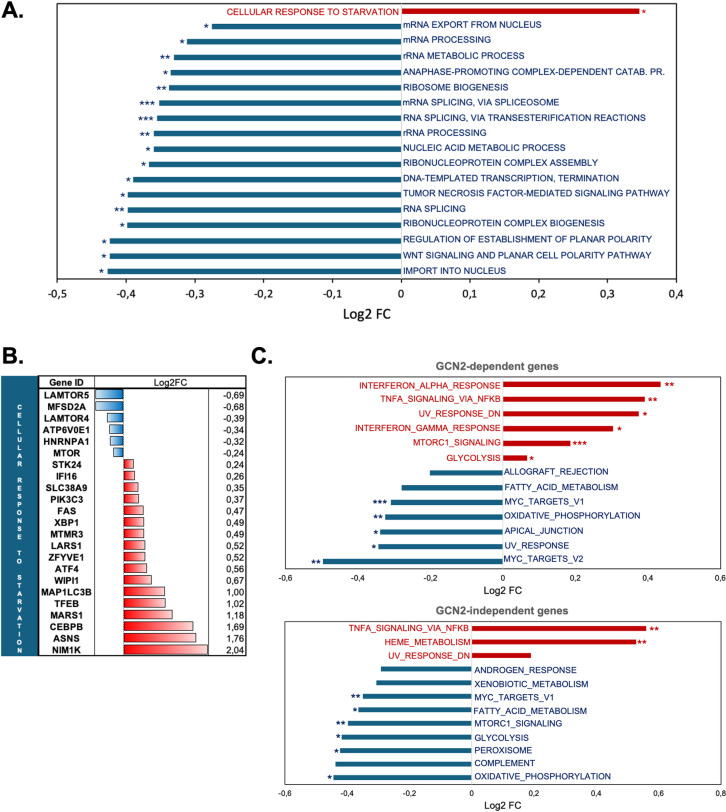
Gene Ontology (GO) and Gene Set Enrichment Analysis (GSEA) of T cells after culture in leucine-deprived media for 6 hours according to GCN2-dependency. **A** GO using GO Biological Process of GCN2-dependent genes. Results of gene set enrichment are expressed in Log2-FC. The number of stars indicates enrichment significance according to meta q-value ( * <  0.05, ** <  0.01, *** <  0.001). Red bar represents positively enriched gene sets (log2-FC >  0) and blue bars represent negatively enriched gene sets (log2-FC <  0). **B.** mRNA expression level of GCN2-dependent DEGs in the “Cellular response to starvation” gene set. Red bars represent up-regulated genes (log2-FC >  0) and blue bars represent down-regulated genes (log2-FC <  0). **C.** GSEA using the Molecular Signatures Database (MSigDB) hallmark gene set collection according to GCN2-depency (up: GCN2-dependent genes; bottom: GCN2-independent genes). Results of gene set enrichment are expressed in Log2-FC. The number of stars indicates enrichment significance according to fgsea q-value ( * <  0.05, ** <  0.01, *** <  0.001). Red bars represent positively enriched gene sets (log2-FC >  0) and blue bars represent negatively enriched gene sets (log2-FC <  0).

Finally, we performed gene ontology (GO) analysis specifically on the dataset of GCN2-dependent genes. GO analysis revealed that most biological processes were negatively enriched, consistent with the stress induced by amino acid deprivation (RNA processing and splicing, ribosome biogenesis, nucleic acid metabolic processes, etc.) (Fig 5A). The “cellular response to starvation” biological process was the only one positively enriched (log2-FC =  0.34616; q-value =  0.038), with 17 upregulated genes out of the 23 presents in our dataset (Figs 5A-B). GSEA analysis (using the MSigDB hallmark gene set collection as previously described) of GCN2-dependent and GCN2-independent genes showed that the “TNFα signaling via NFκB” gene set was positively enriched in both datasets, indicating that only a part of genes of this gene set is dependent of GCN2 kinase (Fig 5C). The “Glycolysis” and “mTORC1 signaling” gene sets were positively enriched in GCN2-dependent genes and negatively enriched in GCN2-independent genes, suggesting they could be dependent on GCN2. The “Interferon responses (α and γ)” also appeared to be dependent on GCN2, as they were not enriched in the GCN2-independent dataset. The “Oxidative phosphorylation” gene set was negatively enriched in both datasets, indicating it is non-dependent on GCN2 (Fig 5C).

## Discussion

Our RNA-Seq analysis of activated human primary T cells subjected to 6 hours of leucine deprivation revealed a significant differential expression of 3,431 genes. We chose a relatively permissive p-value threshold (<0.05) and non-stringent fold-change criteria (fold-change > 1 or < 1, i.e., log2-fold-change > 0 or < 0) to capture the broadest possible view of the impact of leucine deprivation. GSEA analysis showed that short-term leucine deprivation led to a positive enrichment in the “TNFα signaling via NFκB” and “interferon-γ response” gene sets. These pathways are of particular interest in the field of cell therapy, as cytotoxic T cells primarily release TNF-α and IFN-γ to induce tumor cell apoptosis and increase tumor cell sensitivity to perforin/granzyme- and Fas/FasL-mediated cell death [17]. We also observed a positive enrichment in the “unfolded protein response” gene set following leucine deprivation. The unfolded protein response (UPR) is a highly conserved pathway that enables cells to cope with stress in the endoplasmic reticulum (ER) caused by the accumulation of misfolded and unfolded proteins. ER stress can activate the integrated stress response (ISR), which maintains cellular homeostasis in response to various stressors such as amino acid depletion, viral infection, heme deprivation, and ER stress. The ISR can be activated by several kinases, depending on the type of stress, such as PERK (protein kinase RNA-like endoplasmic reticulum kinase), PKR (protein kinase R), HRI (heme-regulated inhibitor), or GCN2 (general control nonderepressible 2), which then phosphorylate eIF2α, promoting ATF4 expression [18]. Since the UPR overlaps with the ISR—both responses being triggered by ER stress and resulting in eIF2α phosphorylation and similar target gene expression—we cannot definitively determine whether the enriched gene set corresponds solely to the UPR or to a more general ISR gene set.

There is limited data in the literature on the role of GCN2-induced ISR in T cells and its relevance to anti-tumor immunity. A recent study by St Paul *et al.* demonstrated that GCN2 activation in CD8 + T cells through amino acid starvation (4 days of arginine starvation) or halofuginone treatment (48 hours) enhances oxidative metabolism and effector function, including increased IFN-γ production. The adoptive transfer of halofuginone-treated CD8 + T cells into tumor-bearing mice resulted in robust anti-tumor responses, suggesting that activating the amino acid starvation response can enhance T cell metabolism and anti-tumor activity [19]. Therefore, activation of the GCN2-ATF4 pathway by short-term leucine deprivation could be advantageous in adoptive cell therapy to promote anti-tumor responses, as prolonged amino acid starvation might be detrimental.

Our analysis with a pharmacological GCN2 inhibitor demonstrated that 60% of the DEGs in our dataset are dependent on GCN2 activation in T cells under leucine deprivation. “Glycolysis” and “mTORC1 signaling” gene sets were found to be GCN2-dependent, as their enrichment patterns differed between the two groups of DEGs based on GCN2 dependency. It has been previously reported that GCN2 regulates glycolysis through the eIF2α-HIF1α-glycolysis pathway [20,21]. GCN2 and mTORC1 both play crucial roles in the regulation of amino acid homeostasis. While GCN2 is activated by amino acid starvation, mTORC1 is inhibited. The interaction between these two signaling pathways remains unclear. It appears that in the context of leucine deficiency, ATF4 contributes to mTORC1 inhibition by upregulating two mTORC1 inhibitors: REDD1 and Sestrin2 [22]. In contrast, there is little data on the role of GCN2 in activating the interferon pathway, although our GSEA analysis showed that “Interferon responses (α and γ)” appeared to be GCN2-dependent, as they were not enriched in the GCN2-independent dataset [23,24]. This result suggests a novel link between GCN2 signaling and immune response pathways, though this requires further investigation.

Finally, regardless of GCN2 dependency, short-term leucine deprivation led to a negative enrichment in “mTORC1 signaling” and “Oxidative phosphorylation” gene sets. While repression of oxidative phosphorylation could be detrimental to CAR-T cells [3,4], repression of mTORC1 signaling could be beneficial. It has been shown that mTOR inhibition promotes the memory T cell phenotype, limits glycolysis, and leads to less-differentiated CAR-T cells [3]. For example, PI3K inhibition in conjunction with BCMA-directed CAR-T cells in Burkitt lymphoma- and multiple myeloma-bearing mice resulted in long-term tumor regression, with an increased frequency of CD8 + CD62L + memory T cells [25]. Similarly, IL-15 signaling, which decreases mTORC1 activity, is associated with metabolic changes in CAR-T cells that can improve anti-tumor activity by preserving their stem cell memory phenotype [5].

## Conclusion

Our transcriptomic analysis of activated human primary T cells revealed that 6 hours of leucine deprivation led to the positive enrichment of pathways that could be advantageous in T cell therapy (TNF-α signaling, Interferon-γ signaling, Integrated Stress Response). The involvement of GCN2 in regulating key metabolic and signaling pathways suggests that controlled activation of this pathway could improve the effectiveness of adoptive cell therapies like CAR-T cells. Despite the major role of GCN2 indicated by our dataset analysis, further investigations are necessary to elucidate the underlying mechanisms. The negative enrichment of mTORC1 signaling could also be favorable for CAR-T cell activity. Combining short-term amino acid starvation with NUTRIREG, a technology for transient gene overexpression, could potentially optimize CAR-T cell efficacy by modulating metabolic pathways and enhancing cytokine secretion. Our findings present a compelling case for the exploration of leucine deprivation as a tool to modulate T cell function using NUTRIREG technology, particularly in the context of adoptive cell therapy. The GCN2 kinase plays a significant role in this process, affecting key pathways like glycolysis, mTORC1 signaling, and cytokine response. Further studies could uncover more detailed mechanisms and validate these findings in therapeutic contexts, potentially leading to novel strategies to enhance CAR-T cell efficacy.

## Supporting informations

S1 Table Lists of the 50 most upregulated genes (fold-change ≥  4.09) and the 50 most downregulated genes (fold-change ≤  0.41) in human T cells after 6 hours of leucine deprivation (RNA-Seq).
(TIF)

S1 Fig Analysis by RT-qPCR of mRNA expression level of 12 genes identified from RNA-Seq analysis after 6 hours of leucine deprivation (donor 1) in T cells collected from another healthy donor (donor 2).
mRNA level was assayed by RT-qPCR and normalized on β-actin mRNA level. Heatmap represents results that are expressed as a mRNA log2-fold-change between leucine-free (6h) and control media (*n* = 3–4 per condition).
(TIF)

S2 Fig Analysis by RT-qPCR of mRNA expression level of 12 genes identified from RNA-Seq analysis after 6 hours of leucine deprivation + /- GCN2 inhibitor (donor 1) in T cells collected from another healthy donor (donor 2). mRNA level was assayed by RT-qPCR and normalized on β-actin mRNA level.
Heatmap represents results that are expressed as a mRNA log2-fold-change between leucine-free (6h) and control media (*n* = 2-4 per condition).
(TIF)
