# Supplementary figures and images for: Transcriptomic analysis of human primary T cells after short-term leucine-deprivation and evaluation of kinase GCN2’s role in regulating differential gene expression
Source: PLoS One. 2025 Feb 18;20(2):e0317505. doi: 10.1371/journal.pone.0317505 (PMC11835326; doi:10.1371/journal.pone.0317505)

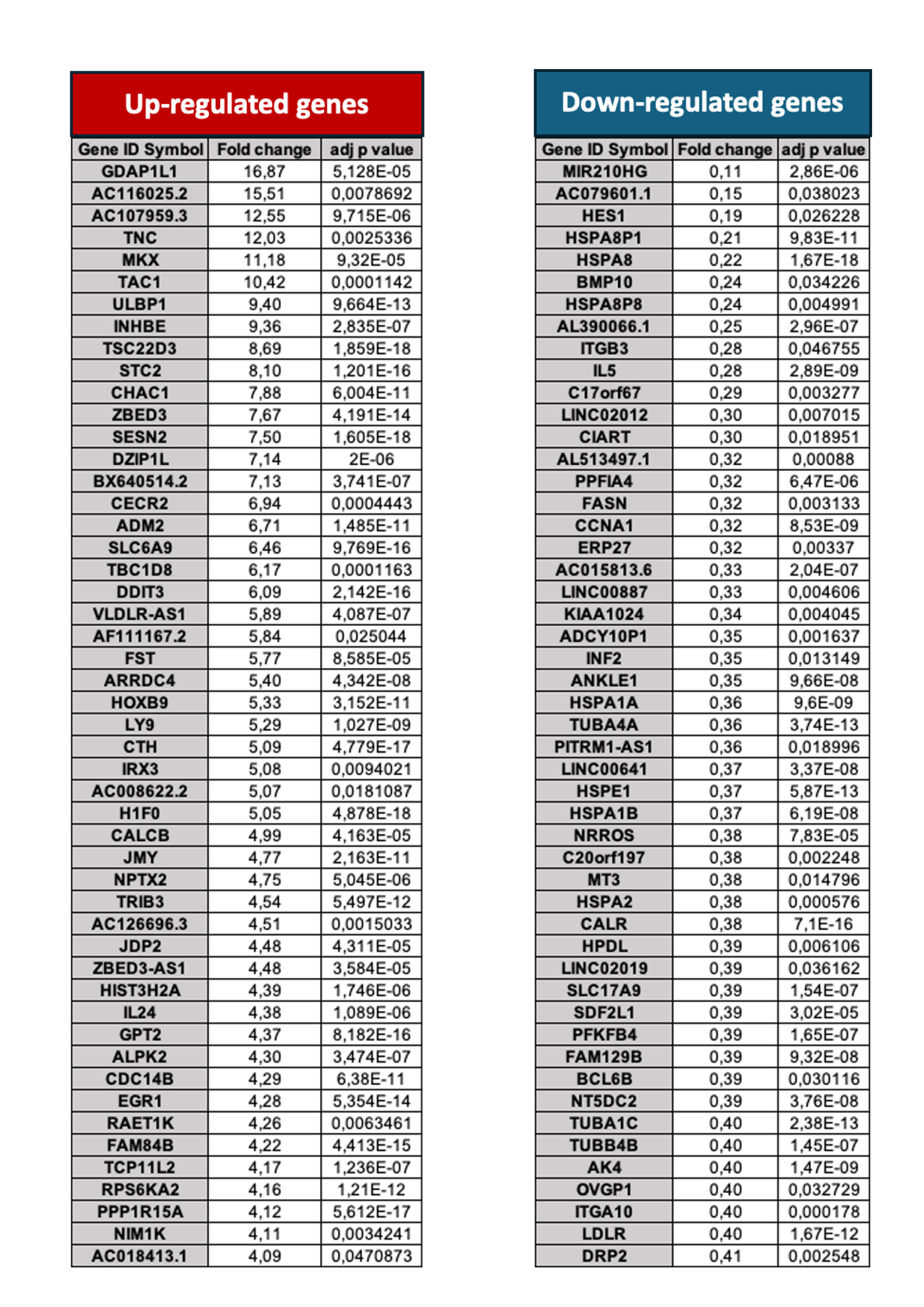

Supplement: S1 Table — (TIF) [file pone.0317505.s001.tiff]

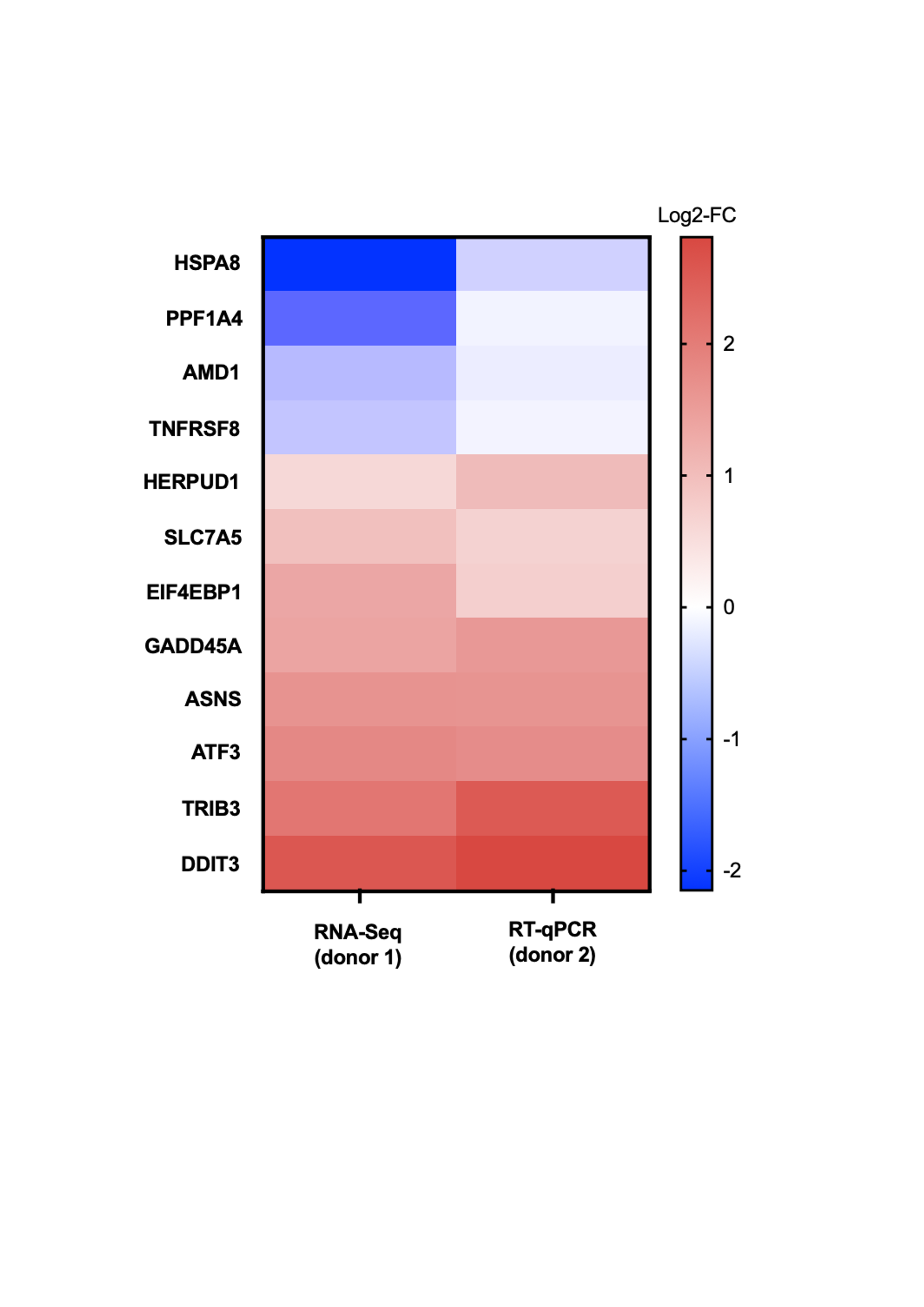

Supplement: S1 Fig — mRNA level was assayed by RT-qPCR and normalized on β-actin mRNA level. Heatmap represents results that are expressed as a mRNA log2-fold-change between leucine-free (6h) and control media (n = 3–4 per condition). (TIF) [file pone.0317505.s002.tiff]
